# Methicillin-Resistant *Staphylococcus aureus* Prevalence among Captive Chimpanzees, Texas, USA, 2012^1^

**DOI:** 10.3201/eid2112.142004

**Published:** 2015-12

**Authors:** Patrick W. Hanley, Kirstin F. Barnhart, Christian R. Abee, Susan P. Lambeth, J. Scott Weese

**Affiliations:** National Institutes of Health, Hamilton, Montana, USA (P.W Hanley);; AbbVie Inc., North Chicago, Illinois, USA (K.F. Barnhart);; University of Texas MD Anderson Cancer Center, Bastrop, Texas, USA (C.R. Abee, S.P. Lambeth);; University of Guelph, Guelph, Ontario, Canada (J.S. Weese)

**Keywords:** Pan troglodytes, methicillin-resistant Staphylococcus aureus, MRSA, veterinary, chimpanzees, Texas, bacteria, antimicrobial resistance, zoonoses

## Abstract

Methicillin-resistant *Staphylococcus aureus* (MRSA) infection in humans and animals is concerning. In 2012, our evaluation of a captive chimpanzee colony in Texas revealed MRSA prevalence of 69%. Animal care staff should be aware of possible zoonotic MRSA transmission resulting from high prevalence among captive chimpanzees.

Methicillin-resistant *Staphylococcus aureus* (MRSA) infection is a threat among humans; ≈80,000 infections and 11,000 deaths occur each year (*1*). MRSA has been also identified in animals of various species, leading to concerns about animal health and zoonotic transmission. Studies have found animal-origin MRSA in humans and human-origin MRSA in animals. Strains of MRSA isolated from companion animals tend to be the same as the strains isolated from humans in the same geographic areas (*2*), and these isolates cluster together even according to highly discriminatory whole-genome sequencing (*3*). In contrast, livestock-associated MRSA strains, which are sequence type (ST) 398, can be found in humans, and animal contact is a well-characterized risk factor for human infection or colonization (*4*).

Although MRSA colonization in companion animals has been established, a paucity of literature exists on MRSA in laboratory animals, specifically those most closely related phylogenetically to humans: chimpanzees (*Pan troglodytes*). Recent studies have demonstrated possible transmission of *Staphylococcus* spp. with no methicillin resistance between sanctuary workers and chimpanzees in Africa (*5*). An additional report from Africa identified human-associated strains of *S. aureus* in captive and wild chimpanzees; some antimicrobial drug–resistant isolates were identified, but oxacillin (methicillin) resistance was not found (*6*). 

At the University of Texas MD Anderson Cancer Center Michale E. Keeling Center for Comparative Medicine and Research in Bastrop, Texas, USA, convenience sampling during physical examinations of chimpanzees revealed increased coagulase-positive *Staphylococcus* spp. resistant to methicillin. On the basis of this information, we prospectively evaluated MRSA prevalence among chimpanzees in this facility, which is accredited by the Association for Assessment and Accreditation of Laboratory Animal Care.

## The Study

At the time of the study, 167 chimpanzees at the facility were housed in male and female groups and had daily indoor/outdoor access. All animals were part of an approved Institutional Animal Care and Use Committee protocol and were managed in accordance with the US Department of Agriculture Animal Welfare Regulations and the Guide for the Care and Use of Laboratory Animals (http://www.nap.edu/catalog/12910/guide-for-the-care-and-use-of-laboratory-animals-eighth). Chimpanzees were observed at least 3 times daily by licensed veterinarians or experienced caretakers, and each year, chimpanzees were sedated and physically examined. 

All chimpanzees were enrolled in a comprehensive training and enrichment program. Positive reinforcement training techniques facilitated voluntary cooperation with daily husbandry or veterinary procedures (*7*). The chimpanzees had an extensive repertoire of trained behaviors including, but not limited to, presenting body parts for inspection and medical treatment, voluntarily presenting legs or arms for intramuscular anesthetic injections, and voluntary submitting to venipuncture (*8*). During this study, the chimpanzees voluntarily presented their faces so that trainers could swab the internal nares. Nasal swab samples were collected for culture from any animals for whom a veterinary examination was scheduled during the collection period; no animals were sedated solely for this study. We tested animals from 18 social groups (4–12 animals/group) that had no between-group physical contact. We excluded 9 chimpanzees that had a history of experimental exposure to hepatitis C virus or HIV. 

Nasal samples for culture were taken with a polyurethane foam swab (CultureSwab EZ Collection and Transport System; Becton, Dickinson and Company, Sparks, MD, USA, and Franklin Lakes, NJ, USA). The swabs were placed in 2 mL of enrichment broth containing 10 g/L tryptone T, 75 g/L sodium chloride, 10 g/L mannitol, and 2.5 g/L yeast extract and were incubated for 24 h at 35°C. Aliquots of 100 µL were streaked onto MRSA chromogenic agar (BBL CHROMagar; Becton, Dickinson and Company) and incubated at 35°C for 48 h. Tube coagulase-positive isolates were identified as *S. aureus* by latex agglutination test (Pastorex Staph Plus; Bio-Rad Laboratories Ltd., Mississauga, Ontario, Canada). Methicillin resistance was confirmed by presence of penicillin-binding protein 2a antigen detected by use of a latex-agglutination test (Oxoid Ltd., Basingstoke, UK). MRSA isolates were characterized by *spa* typing; types were characterized by using the Ridom SpaServer (http://SpaServer.ridom.de) (*9*). Real-time PCR was used for detection of the *luk*F-PV gene encoding Panton-Valentine leukocidin (*10*).

During a 1-month period, samples were collected from 125 chimpanzees and MRSA was isolated from 86 (69%; 95% CI 61%–77%). Three chimpanzees were sampled twice, and results were positive on both occasions, for a total of 89 positive samples. A total of 57 of the 86 MRSA isolates (66%; 95% CI 58%–74%) were positive for Panton-Valentine leucocidin t008, consistent with the ST8 USA300 clone. Most of the remaining isolates corresponded to 6 spa types related to t008: t818 (19 [22%]); t024 (4 [4.7%]); t197 (2 [2.3%]); t2030 (2 [2.3%]); and (1 [1.2%] each t9141, t682, and t6172) (Table). Single isolates of t116 and t1754, related to each other but distinct from ST8, were also found. Of the 3 chimpanzees that were sampled twice, the same strains (t008, t818) were identified in both cultures for 2, and 2 different, but related, strains (t024, t818) were identified in each culture for 1.

**Table:** *spa* types of methicillin-resistant *Staphylococcus aureus* cultured from the nasal cavity of captive chimpanzees that were separated according to sex, University of Texas MD Anderson Cancer Center, Bastrop, Texas, USA, 2012

*spa* type	Chimpanzee sex		Total
	M	F	
t008			
No.	30	27	57
% Within type	52.6	47.4	100
% Within sex	71.4	57.4	64.0
% Of total	33.7	30.3	64.0
T818			
No.	9	11	20
% Within type	45.0	55.0	100
% Within sex	21.4	23.4	22.5
% Of total	10.1	12.4	22.5
t024			
No.	1	3	4
% Within type	25.0	75.0	100
% Within sex	2.4	6.4	4.5
% Of total	1.1	3.4	4.5
T197			
No.	0	2	2
% Within type	0	100	100
% Within sex	0	4.3	2.2
% Of total	0	2.2	2.2
T2030			
No.	1	1	2
% Within type	50.0	50.0	100
% Within sex	2.4	2.1	2.2
% Of total	1.1	1.1	2.2
T9141			
No.	0	1	1
% Within type	0	100	100
% Within sex	0	2.1	1.1
% Of total	0	1.1	1.1
T682			
No,	0	1	1
% Within type	0	100	100
% Within sex	0	2.1	1.1
% Of total	0	1.1	1.1
T6172			
No.	1	0	1
% Within type	100	.0	100
% Within sex	2.4	.0	1.1
% Of total	1.1	.0	1.1
T1754			
No.	0	1	1
% Within type	0	100	100
% Within sex	0	2.1	1.1
% Of total	0	1.1	1.1
Total			
No.	42	47	89
% Within type	47.2	52.8	100
% Within sex	100	100	100
% Of total	47.2	52.8	100

## Conclusions

On the basis of the presence of MRSA in clinical specimens, along with the close contact between animals in the facility, we hypothesized that the prevalence of nasal carriage of MRSA in the chimpanzee colony would be similar to that in high-risk human populations, such as hospitalized patients in long-term care facilities (58%–67%) (*11*). Our finding of nasal carriage of MRSA in 69% (95% CI 61%–77%) of chimpanzees was consistent with that estimate but remarkable and concerning. Limited corresponding data from other facilities that house nonhuman primate species hampers our ability to compare rates. However, the paucity of published data does not indicate that nasal carriage of MRSA in nonhuman primates is rare; anecdotal information suggests that MRSA is widespread in these colonies. The lack of data may result from reluctance to publicize infections, given the sensitivities regarding management of research animals or from not using culture methods to identify MRSA (*12*).

Most isolates were characterized as the USA300/ST8 strain, which is considered a community-associated strain (*13*). The predominance of human epidemic clones of MRSA was not surprising because human strains are found in animals (*14*). However, the predominance of USA300-related strains was noteworthy because in the United States, this strain is most often found in community-associated MRSA infections and the USA100 strain is commonly found in human carriers. Our finding could be the result of a chance entry of those strains into the facility. It is unclear why no USA100 MRSA clones were found. It is possible that USA300 strains are more adept at colonizing chimpanzees. The variety of related strains could reflect long-standing presence of MRSA in the population and gradual genetic variation or repeated introduction of strains. Host tropism of different MRSA strains in chimpanzees warrants further attention.

This level of MRSA positivity is cause for high concern for possible transmission to animal care staff. Among veterinary personnel, rates of MRSA colonization are high (*15*) and exceed rates among their animal patients. On the basis of personal protective equipment use at this facility, we would expect low or absent carriage rates among the animal care staff. Further study of the dynamics of MRSA in nonhuman primate colonies and interspecies transmission is warranted.
